# Preparing for the European Health Data Space: an open-source compiler for fast, transparent, and portable health data transformations

**DOI:** 10.3389/fmed.2025.1661091

**Published:** 2025-09-11

**Authors:** Stefan Beyer, Nikola Tanjga, Gabriel Kleinoscheg, Dieter Hayn, Klaus Donsa, Karl Kreiner, Günter Schreier

**Affiliations:** ^1^Center for Health & Bioresources, AIT Austrian Institute of Technology, Vienna, Austria; ^2^Institute of Neural Engineering, Graz University of Technology, Graz, Austria; ^3^ELGA GmbH, Vienna, Austria

**Keywords:** electronic health record (EHR), interoperability, standards, data transformation, FHIR, FHIR mapping language (FML), MaLaC-HD, European Health Data Space (EHDS)

## Abstract

**Introduction:**

Healthcare systems generate vast amounts of data in diverse and often incompatible formats. Efficient conversion between these formats is essential to ensure interoperability and enable secondary data use, particularly in the context of the European Health Data Space (EHDS) and the proposed Austrian Health Data Donation Space (AHDDS). While standards such as HL7 FHIR aim to facilitate interoperability, inconsistencies in implementation persist. Electronic health record (EHR) providers, including Austria’s ELGA, continue to face challenges in this area. The FHIR mapping language (FML) offers a promising solution for format translation, but current tools for executing FML mappings are limited, especially in terms of processing speed. To address this gap, there is a pressing need for a compiler that translates FML mappings into efficient, executable code.

**Materials and methods:**

We developed the Mapping Language Compiler for Health Data (MaLaC-HD), which compiles FML code into Python. To assess performance, we benchmarked the compiler using a large ELGA document on a typical end-user device, comparing execution speed with existing FML tools. Baseline overhead was measured using an empty mapping. Conformance was manually evaluated by comparing the output of a wide range of example mappings and input data against the Java reference implementation. Additionally, we analyzed the structure and correctness of the generated Python code to assess functional completeness.

**Results:**

After adjusting for overhead, MaLaC-HD achieved execution speeds nearly 100 times faster than existing tools. The output closely matched that of the reference implementation, with only minor discrepancies. The generated Python code met all functional requirements and demonstrated the compiler’s ability to support complex transformations. MaLaC-HD is publicly available under the LGPL license.

**Conclusion:**

MaLaC-HD can serve a wide array of use cases and has the potential to integrate with existing platforms for secondary data use to support large-scale health data research across Europe and beyond. MaLaC-HD could provide the EHR community with a powerful, efficient tool for accelerating data transformation, an essential capability for the success of the EHDS initiative.

## 1 Introduction

Health data is continuously generated across diverse global contexts, including clinical settings, personal health devices, and manual data entry through forms (1). A significant challenge arises from the use of heterogeneous and often incompatible data exchange formats, with some datasets lacking any standardization altogether (2–4). These incompatibilities may stem from the absence of common standards, the use of proprietary formats, or inconsistencies in data representations. While such issues are observed across various domains, they are particularly pronounced in healthcare. Due to regulatory requirements related to data protection, scalability limitations, and prevailing clinical workflows, health data is frequently stored and processed locally (5). Consequently, this results in fragmented data silos that are either weakly interconnected or entirely isolated.

Addressing the challenges of fragmented health data systems requires seamless communication, efficient data exchange, and effective collaboration across heterogeneous environments, all while ensuring security, scalability, and operational efficiency. Enhanced interoperability is anticipated to not only improve patient outcomes, reduce clinical errors, and facilitate collaborative healthcare delivery in primary care settings, but also to enable secondary data use for research and policy-making purposes in the longer term (6–8). Interoperable infrastructures may be implemented at regional or national scales, such as the proposed Austrian Health Data Donation Space (AHDDS) (9), or expanded to European initiatives like the forthcoming European Health Data Space (EHDS) (10) and potentially even to global frameworks. To support this goal, HL7 International introduced the FHIR mapping language (FML) (11) alongside the corresponding StructureMap resource (12). These standards aim to enable the definition and exchange of data transformation mappings in a consistent, reusable format, and are supported by tools executable in both local and cloud environments, such as FHIRPath Lab (13). Currently, FHIRPath Lab supports three transformation engines: HAPI FHIR (14), Matchbox (15), and a .NET-based implementation (16). The HAPI FHIR and Matchbox engines derive from the original Java implementation developed by Grahame Grieve (17), which was subsequently ported to .NET by Brian Postlethwaite. For secondary data use, several tools provide partial or full support for FML functionalities. Examples include Microsoft’s FHIR Anonymization Tool (18), which leverages the FHIRPath submodule (19), and the FHIR to OMOP Implementation Guide (IG) (20), developed under the HL7 Vulcan Accelerator initiative (21), which facilitates the full FML engine.

However, preliminary evaluations indicate that existing tools for executing FML transformations exhibit suboptimal performance, often requiring several seconds to process large EHR documents on contemporary end-user hardware. This latency presents a significant limitation for scenarios involving high-throughput environments, such as linking national infrastructures like Austria’s ELGA system with the EHDS. The performance bottleneck is likely attributable to the fact that current implementations rely exclusively on interpreters, which appear unable to meet the required execution speed. While the development of a low-level virtual machine or bytecode compiler could address these limitations, it is widely acknowledged, including by the developers of existing tools, that such an approach would incur substantial implementation and maintenance costs. Compiler-based solutions demand sophisticated optimizations and complex low-level code generation techniques to achieve acceptable runtime performance (22, 23). In the context of a proprietary product, these requirements would likely translate into significant financial barriers, rendering the solution inaccessible for many implementers or researchers within the EHDS ecosystem. Conversely, there is limited incentive to develop and sustain a high-performance open-source engine, given the long-term commitment required to ensure functional correctness and cybersecurity resilience, even for large organizations. Transparency is also a critical requirement for ensuring traceability, fostering trust, and supporting good research practices in health data processing. However, based on our experience and discussions with experts in this area, current tools often lack sufficient transparency, as their internal mechanisms are typically obscure and difficult to understand. Existing FML engines also exhibit limited flexibility with respect to input and output data formats, as they all depend on the availability of structure definitions (24), a specific metadata format, for all involved models. This introduces considerable overhead, requiring substantial manual effort for the specification and maintenance of metadata models, which becomes particularly burdensome when they are subject to frequent changes. Despite its potential, FML also remains difficult to adopt for individuals and smaller organizations, as our experience suggests.

A potential alternative involves translating FML mappings into an established general-purpose programming language, thereby leveraging the performance, maturity, and ongoing security auditing of existing runtime environments. Modern programming languages benefit from active development communities, frequent security updates, and relatively stable language specifications, which help minimize maintenance costs and promote long-term stability. Additionally, their advanced debugging capabilities can be facilitated, in contrast to the limited debugging support existing tools currently offer. Furthermore, by statically compiling the generated code, the risk of runtime vulnerabilities can be mitigated. Executing simple, transparent mapping code that clearly reflects the original FML definitions, can also support technical verification and validation processes. Also, it is feasible to use readily available resources to automatically generate the necessary input and output models, instead of relying on the manual creation of structure definitions. In generated code, missing functionality that is not covered by FML might also be easily added and even extending the code generation to multiple programming languages should be feasible. This would overcome the limited flexibility and portability of current tools, that are typically bound to a single technology stack and do not support custom logic. Thus, translating FML mappings to a general-purpose language may offer a pragmatic path toward developing a more efficient and sustainable mapping engine.

To mitigate the current interoperability issues in healthcare, the present paper concerns the development of a mapping language compiler that generates fast, transparent and portable code from a standardized mapping language, such as FML.

## 2 Materials and methods

To demonstrate the feasibility of developing an FML compiler, we implemented the Mapping Language Compiler for Health Data (MaLaC-HD) in Python. This prototype was developed following the minimum viable product (MVP) methodology (25) and serves as an FML to Python compiler. The source code is publicly available in the official repository: https://gitlab.com/cdehealth/malac-hd.

### 2.1 Architecture

**FIGURE 1:**
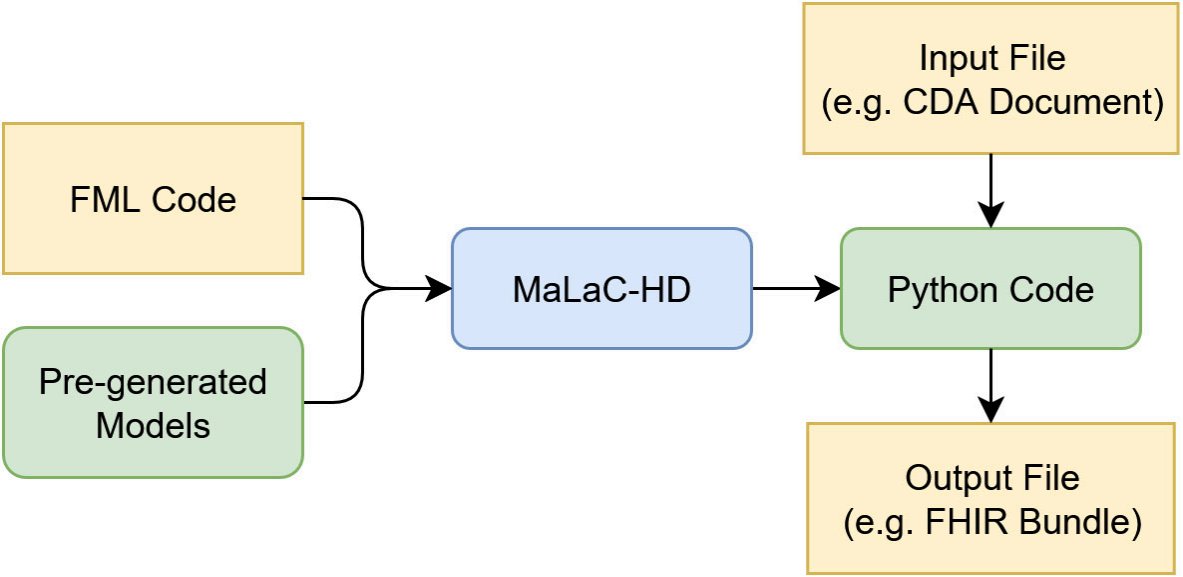
Overall translation process of MaLaC-HD.

**FIGURE 2:**
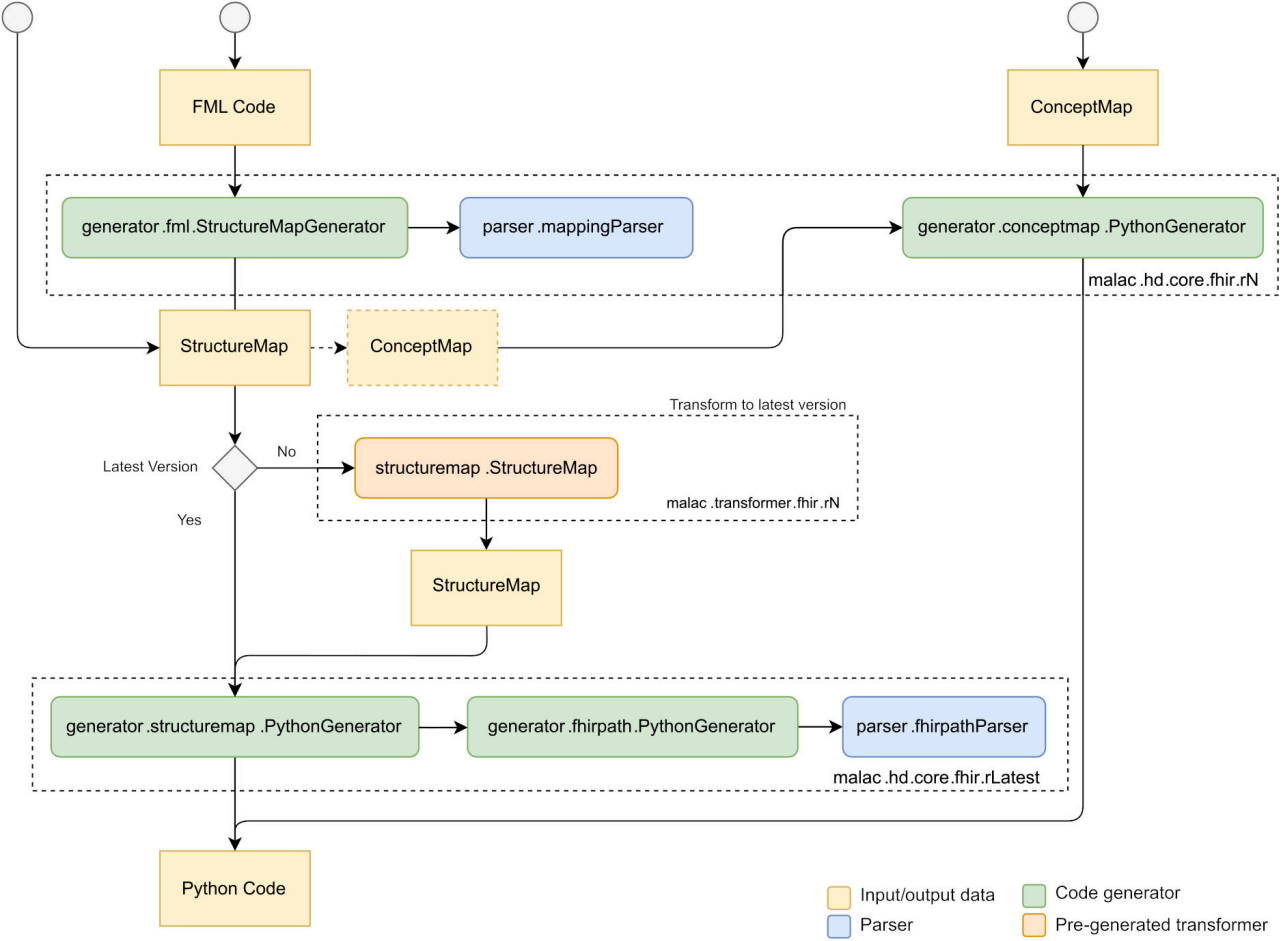
Data and control flow between the components of MaLaC-HD, based on (54).

Figure 1 outlines the overall process of translating and executing mappings. Initially, the FML mappings are compiled into executable Python code. Pre-generated model classes supply the required type information for the source and target data structures. This compilation step is performed only once, provided the underlying mapping logic remains unchanged. Subsequently, the generated Python code utilizes the models to parse the input document, specifically a CDA instance, and performs a direct transformation into the designated target structure, namely a FHIR Bundle. Figure 2 illustrates the typical interaction between the individual components during the translation of FML code to Python. The compiler also supports the translation of StructureMap resources and individual ConceptMap resources (26). ConceptMap resources define the mappings between concepts from distinct terminologies, which is essential when transforming health data between formats that utilize different coding systems, such as LOINC (27) or SNOMED CT (28). A StructureMap may contain embedded ConceptMap resources or reference external ConceptMap resources. Conversely, FML supports the representation of simple concept maps within a StructureMap and allows references to external ConceptMaps, thereby providing flexible mechanisms for terminology mapping within data transformations. Internally, the compiler only implements the latest FHIR version for the StructureMap generator. However, by utilizing pre-generated transformers, created using the compiler itself and derived from official mappings, it can parse FML and/or StructureMap resources across multiple FHIR versions. Presently, the compiler supports versions R4 and R5. Support for newer versions might be added by incrementally porting the existing codebase and incorporating transformers from previously supported versions into the subsequent releases.

For the evaluation of FHIRPath expressions, the StructureMap generator employs another dedicated Python generator. Since StructureMap resources retain raw FHIRPath expressions as strings, these expressions are parsed only at this stage. We have successfully implemented a single-pass approach that generates concise, optimized Python code expressions on-the-fly during the parsing of each FHIRPath string, thereby improving both readability and execution efficiency.

Finally, the generated Python code can be executed directly as a standalone script or integrated as a module within existing Python applications.

### 2.2 Implementation

As initial use cases, we selected the conversion from CDA to FHIR, as well as transformations between FHIR versions R4 and R5. This choice is motivated by the fact that several national EHR providers within the European Union, including Austria’s ELGA system, currently utilize CDA for medical data representation (29). Conversely, the European Health Data Space (EHDS) regulation promotes FHIR for newly defined document types, necessitating data transformations to FHIR in multiple jurisdictions in the near future (30). Even in settings where FHIR is already adopted, interoperability challenges persist due to the coexistence of different FHIR versions, requiring reliable mechanisms for version-to-version data conversion.

#### 2.2.1 MVP methodology

The development of all MaLaC-HD components adhered to the MVP methodology, iteratively applying the build-measure-learn cycle. MVPs were rapidly constructed to evaluate key hypotheses, including improved execution speed and enhanced debuggability. Early feedback collection prior to implementing additional features allowed for timely assessment of progress toward these objectives, minimizing resource expenditure. This iterative process facilitated continuous learning through empirical insights, enabling rapid identification and integration of improved solutions as necessary.

#### 2.2.2 Data models and parsers

We employed established tools such as generateDS (31) to automatically generate source and target data structures as native Python classes. The official XML schemas for CDA and FHIR served as input for this process. To resolve data types that could not be inferred directly from the mapping code, we utilized Python’s code introspection capabilities on the generated classes. This required augmenting the generated code with type hints, a feature not originally supported by generateDS. Additional enhancements included resolving conflicts arising from identical namespace prefixes used in included and imported schemas, as well as introducing aliases for elements belonging to namespaces different from the default namespace.

The FML parser was introduced subsequently as an optional component of the compiler, given that it is not central to our primary hypothesis, as alternative tools are available to convert FML into StructureMap resources. Nevertheless, because FML may contain embedded FHIRPath expressions, which are preserved in their raw form within StructureMap resources, an additional parser was required. Official ANTLR4 grammar files for both FML and FHIRPath were available (32), and required only minor modifications to enable Python code generation using the standard toolchain. Specifically, certain grammar rules were adjusted with regards to certain keywords and escape sequences, ensuring accurate parsing of all tested FML files. Leveraging these existing grammar definitions facilitated rapid implementation of both parsers.

To support JSON serialized FHIR resources, we extended generateDS with a generic JSON export functionality. By introducing several additional method overloads in the generated code, we achieved a fast and standards-compliant FHIR JSON serialization. For JSON import, dynamic code introspection was employed to parse the JSON data and instantiate the corresponding data classes in memory.

#### 2.2.3 Code generators

The code generator was divided into two components: the StructureMap generator, implemented initially, and the FHIRPath generator, introduced subsequently. This modular approach enabled incremental testing of our hypotheses. Initially, we focused exclusively on FML code without embedded FHIRPath expressions. Where necessary, a prototypical FHIRPath translation was incorporated, substituting a limited set of well-known FHIRPath expressions with equivalent Python code. Given that most commonly used FML expressions are relatively straightforward to implement, this strategy facilitated early validation of our hypotheses without the immediate need for comprehensive FHIRPath support.

For the initial implementation of the FHIRPath generator, we produced verbose but semantically equivalent Python code, incorporating helper functions to avoid bloated code. Subsequently, to enhance code readability and reduce verbosity, we introduced optimizations that identify and replace common subexpression patterns with more efficient alternatives during code generation. This approach enabled single-pass Python code generation without requiring an intermediate representation. Maintaining a simple generator design was a deliberate choice to facilitate future extensions, allowing straightforward adaptation for additional target programming languages.

The FML generator underwent iterative refinement throughout development. Substantial effort was dedicated to enhancing type handling. To avoid reliance on an additional intermediate data model, as employed by other tools, we directly managed the mapping of various XML data types to corresponding CDA and FHIR types during the translation process. This remains an ongoing effort aimed at achieving comprehensive type support across all supported CDA, FHIR, and XML data types. Furthermore, compatibility with alternative data models, such as OMOP and JSON, was explored to demonstrate the compiler’s extensibility and adaptability.

### 2.3 Evaluation

The desired properties of the compiler were evaluated using a diverse set of test mappings. Performance and conformance assessments primarily centered on the laboratory report example. For the evaluation of other properties, additional mappings were employed to demonstrate their feasibility.

#### 2.3.1 Conformance

The Java implementation employed by HAPI and Matchbox was used as the gold standard for conformance evaluation. Conformity was assessed by comparing test outputs using diff tools, permitting only valid differences such as automatically generated random identifiers, whitespace variations, and other minor discrepancies arising from imprecise specifications.

To achieve broad coverage of mappings, we developed several mappings internally and incorporated multiple publicly available mappings with corresponding example documents. Initially, we created a simple handcrafted FML map, followed by an early version of the CDA to FHIR conversion for the ELGA laboratory report (33), utilizing the official example report (34), and building upon HL7 Suisse’s work (35). Additionally, we incorporated the publicly available Italian and Swiss CDA models and transformations (36–38), with the latter including FHIR-to-CDA conversions. Subsequently, we integrated R4 and R5 conversions from the FHIR specification (39), which required some corrections and adaptations to function properly. These conversions also maintained compatibility with R4 input maps, after our migration of the core system to R5 during development. For the FHIRPath submodule, we incorporated the official test suites for both R4 and R5 (40), which cover complex features such as functions, aggregations and type coercion. As our focus was on the transformation process rather than validation, all tests were conducted using valid FML maps and input data.

#### 2.3.2 Performance

Performance testing of the mappings was conducted locally, with the execution speed of Matchbox serving as the reference benchmark. The laboratory report maximum example provided by ELGA was selected due to its complexity and realistic representation. Additionally, a minimal example lacking transformation instructions was created to estimate baseline overheads.

Benchmark executions were performed on a standard Dell Latitude 5450 laptop with the following specifications:

- Intel^®^ Core™ Ultra 7 165H 3.80 GHz CPU
- 32,0 GB 5600MHz RAM
- BG6 KIOXA 1024GB SSD

The benchmarks were conducted within the Windows Subsystem for Linux (WSL) running an Ubuntu environment. The Matchbox server was deployed in a Docker container. The following software versions were utilized:

- Windows 10 Enterprise, 22H2, Build 19045.5737
- WSL Ubuntu 22.04.5 LTS
- Docker version 28.1.1, build 4eba377
- OpenJDK Runtime Environment (build 21.0.7+6-Ubuntu-0ubuntu122.04)
- Python 3.10.12

The following versions of the FML tools were used to run the benchmarks:

- MaLaC-HD 1.1.0 dev build 721d0623ad350ae19825841ac2ebb4e0da3289ad
- Matchbox v4.0.4 with-cda container configuration

The benchmarking protocol included a warm-up phase of five cycles, followed by a test phase comprising fifty cycles. The corresponding source code is available in a dedicated repository (41).

To account for potential overheads, such as garbage collection cycles, spawning new MaLaC-HD processes, or establishing HTTP connections via Matchbox’s FHIR REST API, we designed a minimal baseline test. This test involved loading and sending input data without performing any transformations. Additionally, the MaLaC-HD benchmark was conducted in multiple modes: running within the same process as the benchmarking script (*inproc*), including an explicit full garbage collection cycle during transformation (*inproc-gc*), and spawning a new child process for each run (*exec*).

To assess potential differences between the two supported output formats (JSON and XML), each benchmark was executed twice, once for each format.

#### 2.3.3 Flexibility

To demonstrate the flexibility of our methodology and assess its applicability to the OMOP CDM, we employed PostgreSQL (42). It was chosen for its open-source license and its capability to export XML schemas as well as XML data from standard database tables. The tables were created based on the official OMOP CDM version 5.4 SQL schema (43). Subsequently, we exported the schema and table data as XML. Due to limitations in the PostgreSQL version used, it was not possible to export all tables simultaneously within the tablespace. Therefore, only a subset of tables relevant to the available test code was exported. We utilized test cases from the Vulcan FHIR to OMOP Implementation Guide (IG), supplemented with an additional test for observations (44), reflecting a use case in the Smart FOX project. Following the model class generation, some adjustments were necessary to the data type handling logic. However, we successfully produced compliant OMOP data outputs. Given the limited number of SQL data types, we anticipate that extending support to all OMOP tables, and more broadly, to various SQL-based databases from different vendors, will require minimal additional effort.

#### 2.3.4 Platform-independence

To demonstrate the compiler’s ability to generate platform-independent code, we executed the generated mappings directly within a web browser. This was implemented as part of the VIDi project (45), which provides an intuitive visualization for the international patient summary (IPS) (46). The VIDi FML code defines rules that specify how display elements and text from the source FHIR bundle should be arranged within the output JavaScript code. The FML can be modified directly to accommodate specific requirements, such as adapting to national IPS variants or adding new features. Traditional transformation methods, including XSLT or preprocessing with Java or Python, as previously employed by ELGA, often raised security concerns. To mitigate these issues, we leveraged PyScript, an in-browser Python runtime, to securely process the VIDi FML. This approach enables users to drag and drop files into any modern web browser and instantly visualize IPS data without the need for additional software.

To evaluate the feasibility of directly generating JavaScript code, we tested several JavaScript libraries capable of importing and exporting XML-based data structures. Our primary focus was on XML import, as the JavaScript output is expected to be JSON-based. Promising libraries such as xml-js and xml2js were examined, both of which allow XML data import without requiring a schema or additional metadata. The main limitation of these libraries is the complete lack of built-in validation. However, alternative tools are available for this purpose.

#### 2.3.5 Transparency, adaptability and debuggability

**FIGURE 3:**
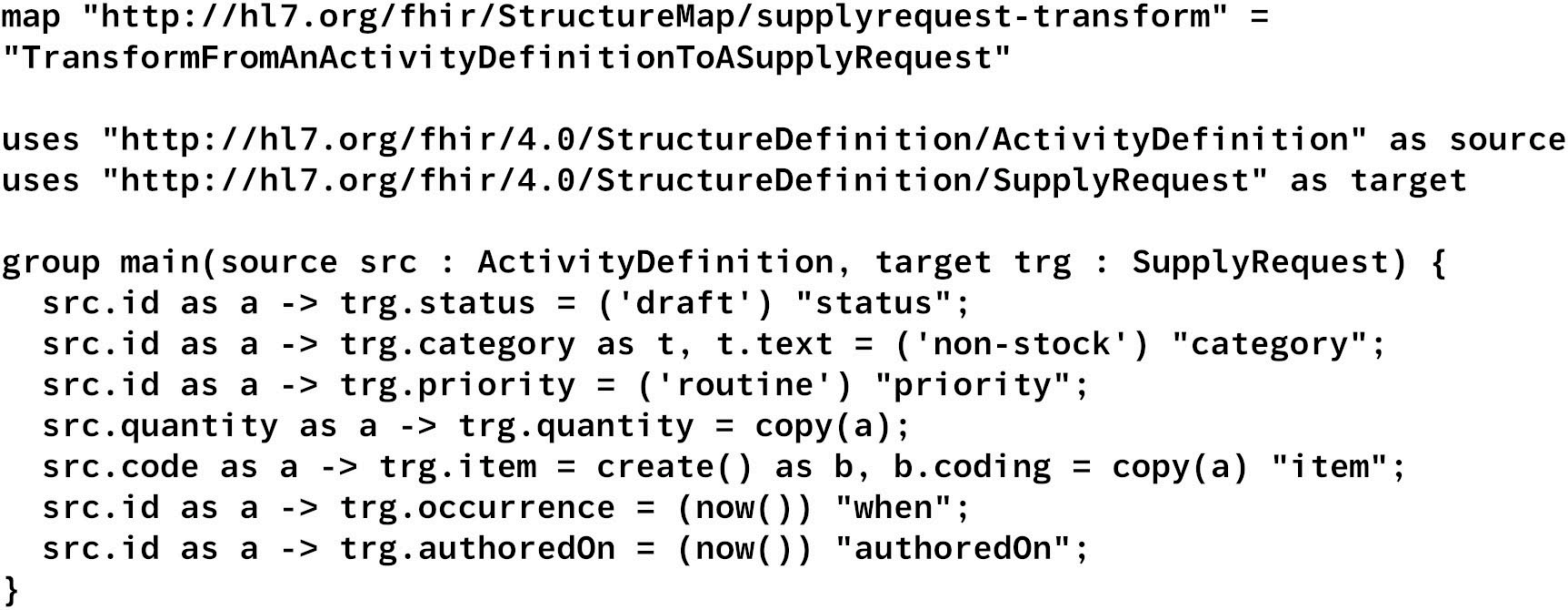
Adapted example FML code.

To demonstrate the correspondence between the original FML code and the generated Python code, we utilized an official example transformation converting an ActivityDefinition to a SupplyRequest, which encompasses a variety of operations (47). As the example contained some discrepancies with respect to the latest FML specification, such as missing parameters in the copy operation, we made slight adaptations, as illustrated in Figure 3. For example, the parameters for the *copy* operation were added. This FML code was subsequently translated into Python using the latest version of the compiler, serving as an example to illustrate transparency, adaptability, and debuggability. Debuggability was also assessed throughout the compiler’s development using various FML code examples. To identify common FHIRPath expressions suitable for simplification, additional code samples were also utilized.

## 3 Results

We successfully created a compiler that can be installed and run with just two simple commands using the packaged version available on PyPI, assuming the Python environment is already configured:

pip install malac-hd

and e.g.,

malac-hd -m CdaToBundle.4.map -co cdaToBundle.py

The translated mapping can then be executed in one single command, e.g.:

python cdaToBundle.py -s LabReport.at.cda.xml -t bundle.4.fhir.xml

Setting up the environment on Windows currently involves running the standard Python installer provided by the Python Software Foundation. On Unix-based systems, setup may vary depending on the distribution and typically includes installing the necessary Python packages and/or creating a virtual environment.

To keep the generated mapping code clean and avoid requiring installation of the full engine just to execute mappings, we separated the runtime dependencies. This approach also enables managing the generated models and commonly used pre-generated transformations in separate repositories. These packages are named *malac-models-cda*, *malac-models-fhir*, and *malac-transformer-fhir*.

Additionally, we created a package called malac-tools, which serves two main purposes. First, it manages FHIR type detection for both source and target models, eliminating the need for any logical or additional meta models. Second, it helps keep the generated FHIRPath code concise and readable by avoiding excessive code duplication. Typically, this package is required only during the FML translation phase, but it may also be executed dynamically during mapping—for example, to handle certain FHIRPath expressions involving dynamic typing. It also acts as a fallback when types cannot be determined statically during translation.

### 3.1 Conformity

Except for some tests covering non-essential functions like unit conversions, all FHIRPath tests passed successfully and produced identical results. Similarly, the FML outputs were consistent with the reference implementation. Minor differences occurred in areas where the FML and FHIR specifications lacked clarity, such as data type casting. These cases have been documented as issues in the repository.

### 3.2 Performance benchmarks

The performance benchmarks were conducted on a single piece of hardware, as detailed in the “2 Materials and methods” section. When the benchmarks were repeated, the results remained consistently stable, as demonstrated below.

As shown in Table 1, the mean runtimes of MaLaC-HD are significantly lower across all execution modes compared to the Matchbox server. Additionally, runtime variation is minimal when MaLaC-HD runs within the same process. Examining our minimal example without any transformations (Table 2) reveals notable increases in runtime when forcing a full garbage collection cycle or spawning child processes, relative to the other modes. Conversely, running MaLaC-HD in the same process results in a statistically significant yet minor runtime difference compared to Matchbox.

**TABLE 1:** Runtimes in seconds for the laboratory report example.

Laboratory report	Mode	Mean	Min	Max	Median	Std
MaLaC-HD	*inproc*	**0.079**	0.067	0.381	0.071	0.046
	*inproc-gc*	0.747	0.587	4.239	0.681	0.493
	*exec*	0.493	0.447	4.002	0.456	0.353
Matchbox	–	**3.506**	2.976	6.770	3.163	0.980

The bold values used for comparison are highlighted.

**TABLE 2:** Runtimes in seconds for the minimal example to estimate the overheads.

No transforms	Mode	Mean	Min	Max	Median	Std
MaLaC-HD	*inproc*	**0.045**	0.035	0.221	0.037	0.029
	*inproc-gc*	0.563	0.422	3.965	0.494	0.482
	*exec*	0.487	0.399	3.941	0.417	0.493
Matchbox	–	**0.073**	0.036	3.357	0.039	0.330

The bold values used for comparison are highlighted.

Examining the results in Table 1 and factoring in the estimated overheads from Table 2, it is evident that compiling mapping code into general-purpose programming language code reduces the mean execution time for transformations by nearly two orders of magnitude. Accounting for overhead, our compiler completes the transformation in approximately 0.034 s on average, whereas Matchbox requires about 3.433 s for the same task.

**FIGURE 4:**
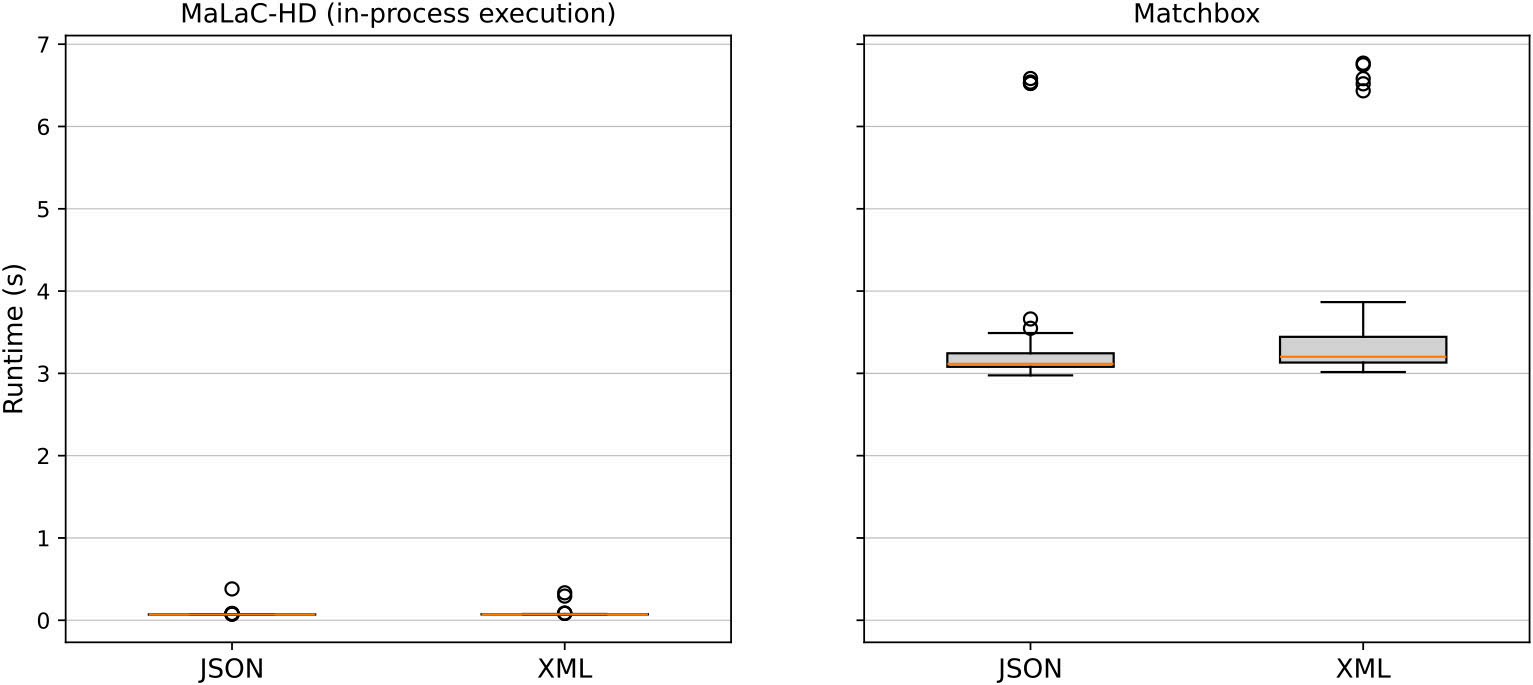
Runtime comparison of the supported output formats for both tools.

When comparing the two supported output formats of our compiler (Figure 4), we observe only insignificant differences between them for both tools, although the variation is higher for the XML output in Matchbox, compared to the JSON output.

### 3.3 Generated code

**FIGURE 5:**
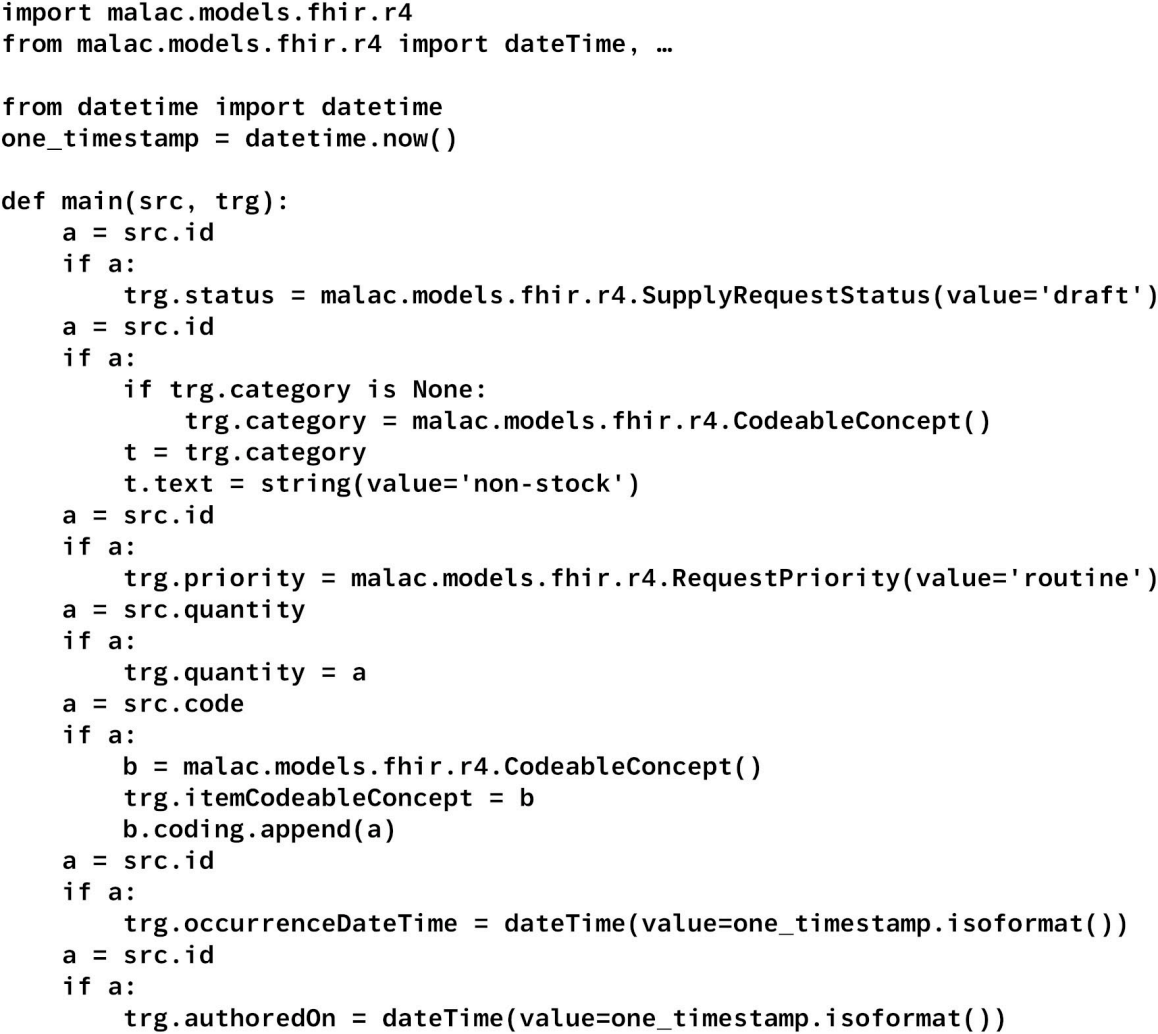
Generated Python code without boilerplate code and superfluous imports.

Figure 5 depicts an excerpt of the Python code, generated from the FML code described in the “2 Materials and methods” section (Figure 3). Note that the complete Python code includes additional imports and boilerplate code. As shown, the Python method is named identically to the corresponding FML group. Parameter and variable identifiers are preserved to maintain traceability. FHIRPath expressions, such as now() and string literals like “routine,” are translated into equivalent Python constructs and optimized for conciseness. Specifically, the now function is evaluated once per mapping execution to generate a single timestamp instance, ensuring deterministic behavior across all references within that mapping. String literals are directly embedded as Python string objects and utilized within the constructors of complex data types or FHIR resources, thereby minimizing extraneous code and improving readability.

The figure further demonstrates that the generated Python code is straightforward to adapt and debug. By preserving identifier names from the original FML wherever feasible, it facilitates easy navigation and correlation between the source FML and the corresponding Python code segments. This supports targeted insertion of additional code, as well as breakpoints during debugging, leveraging standard Python debugging tools to efficiently diagnose issues. Notably, rule names, such as “status” are currently excluded from the generated code to illustrate that their omission does not compromise traceability.

## 4 Discussion

We have designed and implemented a mapping language compiler that translates FML mappings into Python code. Based on benchmarking results and insights gained throughout development, we have demonstrated that our compiler satisfies the following key properties:

- Fast: On the test system, the execution time for the maximum laboratory report example, excluding overhead, was reduced by approximately two orders of magnitude. To estimate overhead, we executed the same example without any mapping logic, thereby isolating factors such as network latency, operating system and runtime initialization delays, and CDA document parsing. Although this approach does not reflect the total end-to-end transformation time, it effectively removes performance variability unrelated to the core mapping logic. Notably, when run within the same process, even the highest recorded runtimes, including overhead, remained below the Doherty threshold of 400 ms (48) for this complex example. These results strongly indicate that the generated code is suitable for real-time, on-the-fly transformations, consistent with the intended use case of ELGA data processing. Furthermore, this performance profile supports scalable bulk transformation of large datasets, which may be necessary for research or analytical applications.
- Transparent: The generated Python code maintains a clear correspondence with the original FML source, enabling straightforward traceability without requiring additional metadata. While embedding the original FML code as comments could further enhance readability and debugging, this feature could be optionally enabled via a compiler flag. Although opportunities for code optimization exist, such as eliminating redundant conditional statements, some degree of duplication is deliberately preserved to keep the generated Python closely aligned with the original FML logic, facilitating transparency and ease of maintenance.
- Flexible: Support for CDA and FHIR R4/R5 was implemented by utilizing generateDS in conjunction with the official XML schemas. This strategy was selected over the use of StructureDefinition resources, commonly employed by other FML engines, due to its enhanced flexibility in accommodating new or evolving data structures. Whenever an XML schema, or a format convertible to XML schema, is available, these definitions can be directly leveraged, enabling immediate support for CDA without incurring additional overhead. Furthermore, the generated data structures proved sufficiently versatile to parse the mapping code itself, as FML is officially represented in FHIR through the StructureMap resource. OMOP support was demonstrated using a manually exported PostgreSQL XML schema. This process can be readily automated via scripting, thereby extending applicability to any tabular data format exportable from SQL databases. Additionally, a model was developed for VIDi, capable of execution within web browsers via PyScript.
- Easy to debug: Standard debugging techniques, such as setting breakpoints, can be employed to analyze the execution of the generated code. Because the generated Python code closely corresponds to the original FML source, errors in the FML mappings can be rapidly identified during development. Conversely, reviewing the generated Python code enables interoperability experts to refine and optimize the original FML code, resulting in more accurate and precise transformations.
- Easily adaptable: We investigated multiple approaches to extend mapping functionality without modifying the FML compiler itself. Presently, our tool generates procedural code, which can be manually adjusted post-generation and managed using standard software version control systems. Using appropriate branching strategies regeneration of code can be facilitated, in case adaptations of the FML code are necessary. Implementing support for object-oriented code generation is expected to be straightforward by adapting the existing procedural code generator. By employing established object-oriented design patterns, such as hooks or interceptors, software developers could modify the behavior of the generated code dynamically, thereby enabling customization without altering the generated source directly.
- Platform-independent: Currently, the compiler exclusively generates Python code. However, extending support to Java or .NET is anticipated to be straightforward, given that model classes with type annotations are already generated from XML schemas and both languages offer robust XML processing capabilities. Regarding JavaScript, we evaluated several XML libraries, some of which appear suitable for handling input and output models even without generated model classes. Furthermore, adding support for lower-level languages such as Rust should be feasible, provided adequate XML processing libraries are available.

Despite promising results, several limitations and areas for further investigation remain:

- Comparative benchmarking: Current performance evaluations have only compared MaLaC-HD against Matchbox, primarily due to its ease of setup. However, other implementations such as HAPI FHIR or the Java command line tool may exhibit different characteristics and runtime overheads. Comprehensive benchmarking across multiple implementations is necessary to contextualize our performance findings.
- Runtime variability: The current comparison is based on only one Python and Java runtime, respectively. For a more comprehensive assessment, it would be beneficial to compare against the .NET implementation to account for differences in JIT compilation strategies and runtime optimizations. Performance may also vary significantly across different JVM versions and vendors, e.g., OpenJDK vs. Oracle JDK. Future evaluations should systematically explore this variability to identify potential performance bottlenecks or optimizations.
- Language and platform constraints: Currently, the compiler only generates Python code. Python was chosen for rapid prototyping and accessibility and is already able to fulfill the performance requirements. Generating lower-level code like Rust or platform-native code like JavaScript could yield further performance improvements, but would have required additional effort. While the generated code might be executed on various platforms, the translation process itself depends on a Python runtime environment and relies on native extensions such as lxml. This precludes compatibility with environments like Jython, which lacks support for Python 3 and native C extensions. Future work may explore modularization strategies to isolate platform-specific components.
- Model coverage: Although the tool currently supports CDA with multiple national extensions and FHIR R4/R5, including profiles and extensions, other widely used data models, such as OMOP or additional SQL-based schemas, are only partially supported or still under development. Also, JSON-based data formats are currently only supported if a corresponding XML schema is present, which is rarely the case. This should be addressed in further iterations of the tool. While CDA transformation capabilities are functional, not all features or edge cases have been fully validated. Additional empirical testing across a wide range of CDA documents, including additional extensions, is needed to ensure robustness and conformity.
- Licensing considerations: The current use of the LGPL license fosters open-source collaboration while permitting integration into commercial systems. However, alternative licensing models such as the Apache License 2.0 may offer broader industry adoption. This trade-off requires further legal and community discussion.
- Evolving debuggability landscape: Although the generated Python code is transparent and can be debugged using standard tooling, other FML engines are actively improving their own introspection capabilities. For instance, ongoing work on traceability features in FHIRPathLab may enhance the debuggability of competing tools, potentially narrowing this advantage. Also, users need to be familiar with Python and it’s debugging capabilities, which might not be always the case.
- Adaptability trade-offs: While the ability to adapt generated procedural code is beneficial for rapid prototyping and customization, frequent manual changes can introduce maintenance overhead and reduce reproducibility. Mechanisms for controlled code extension e.g., through hooks, interceptors, or plugin architectures may help mitigate this issue in future iterations.

### 4.1 Future applications

Through community feedback collected during the software development process, we identified the following non-exhaustive list of potential applications:

- Health data space interoperability: The tool could facilitate efficient integration of regional or national healthcare providers, as well as local EHR systems, with the EHDS. By incorporating pseudonymization techniques, such as EUPID (49), masketeer (50), or FHIRPath-based methods like Microsoft’s Health Data Anonymization tools, it may also support secondary uses, including health data research within frameworks like the AHDDS. Furthermore, these privacy-preserving technologies could prove valuable in enabling data sharing across international partners subject to diverse regulatory jurisdictions.
- IPS visualization: JavaScript code generation may enable in-browser visualization of the IPS, facilitating a unified and standardized approach to IPS presentation across the EHDS and potentially on a global scale.
- FML creation tools: MaLaC-HD could be integrated in visualization tools (51), or testing platforms such as FHIRPath Lab to enable direct and efficient execution of mappings on large datasets interactively, thereby providing immediate feedback.
- Patient-reported outcomes: data exports from cloud platforms such as Apple Health Kit (52), could be shared directly from a web browser or third-party applications, eliminating the need for deep integration via SDKs. Pseudonymization transformations could be performed locally on users’ devices, obviating the requirement for additional app installations.
- Explainable AI mappings: FML and the underlying StructureMap resources could potentially be generated using LLMs or other AI-based tools, leveraging example data or metadata exports such as XML Schemas. Initially, such automatically generated mappings could serve as a starting point for interoperability experts, who would refine them as necessary. Given that visual representations of FML can be interpreted by clinicians, while the corresponding generated code is accessible to software engineers, this approach could eventually enable semi- or fully automated mapping generation with end-to-end traceability. Despite the automation, the mapping process would remain transparent, debuggable, and adaptable, eliminating the need for manual authoring and extensive cross-disciplinary collaboration at every step.
- Commercial applications: By adopting a permissive open-source license such as the LGPL (53) and ensuring broad platform compatibility, the tool may facilitate adoption by industry partners. This, in turn, could encourage contributions to the ecosystem, whether in the form of feedback, financial support, or human resources, while maintaining openness and community-driven development.

As suggested by the use cases outlined above, the developed tool presents a wide range of potential applications, many of which are directly relevant to the forthcoming EHDS. In particular, with the EHDS enabling secondary use of health data, and based on preliminary insights gained during the conceptualization of the AHDDS, there is strong potential for integrating the tool within privacy-preserving infrastructures and explainable AI workflows. Embedding MaLaC-HD into such an ecosystem could reinforce patients’ rights to data access, privacy, and transparency across the European Union, while simultaneously enabling researchers to extract actionable insights to enhance clinical care and inform health policy. Crucially, the open and transparent nature of the transformation process would offer an affordable and trustworthy alternative to opaque and potentially unreliable AI-driven transformation methods. This approach would mitigate the risks of data breaches or loss, particularly in the context of sensitive and structured health data.

## 5 Conclusion

In this work, we have demonstrated the feasibility of developing an open-source mapping language compiler that fulfills a broad set of design requirements, including performance, transparency and portability. Our implementation, developed in Python using the MVP approach, specifically targets the increasingly relevant CDA to FHIR conversion scenario, which is central to both primary and secondary data use in the EHDS for many countries. Benchmarking results confirmed a nearly 100-fold reduction in execution time for our test case compared to existing solutions, without compromising conformity or correctness. A substantial subset of FML operations, including the embedded FHIRPath expressions, has been implemented, omitting only a few non-essential functions.

Future work will involve extending the compiler to support additional target programming languages such as Java, .NET, and JavaScript, conducting more comprehensive performance evaluations across a variety of hardware and software environments, incorporating support for country-specific CDA extensions and expanding compatibility with alternative input and output formats, such as OMOP. Moreover, we aim to explore integration with external services, including pseudonymization pipelines, full-text processing tools, and AI-assisted mapping generation.

## Data Availability

The raw data supporting the conclusions of this article will be made available by the authors, without undue reservation.
